# Ex vivo and in vivo T cell-depleted allogeneic stem cell transplantation in patients with acute myeloid leukemia in first complete remission resulted in similar overall survival: on behalf of the ALWP of the EBMT and the MSKCC

**DOI:** 10.1186/s13045-018-0668-3

**Published:** 2018-10-20

**Authors:** Florent Malard, Myriam Labopin, Christina Cho, Didier Blaise, Esperanza B. Papadopoulos, Jakob Passweg, Richard O’Reilly, Edouard Forcade, Molly Maloy, Liisa Volin, Hugo Castro-Malaspina, Yosr Hicheri, Ann A. Jakubowski, Corentin Orvain, Sergio Giralt, Mohamad Mohty, Arnon Nagler, Miguel-Angel Perales

**Affiliations:** 1 Service d’Hématologie Clinique et Thérapie Cellulaire, AP-HP, Hôpital Saint-Antoine, Paris, F-75012 France; 2 INSERM, Centre de Recherche Saint-Antoine (CRSA), Sorbonne Université, F-75012 Paris, France; 3 Adult Bone Marrow Transplantation Service, Memorial Sloan Kettering Cancer Center, New York, NY USA; 4 Department of Medicine, Weill Cornell Medical College, New York, NY USA; 5 Programme de Transplantation & Therapie Cellulaire, Centre de Recherche en Cancérologie de Marseille, Institut Paoli Calmettes, Marseille, France; 6 University Hospital, Hematology, Basel, Switzerland; 7 Bone Marrow Transplant Service, Department of Pediatrics, Memorial Sloan Kettering Cancer Center, New York, NY USA; 8 Department of Pediatrics, Weill Cornell Medical College, New York, NY USA; 9 CHU Bordeaux, Hôpital Haut-leveque, Pessac, France; 10 Stem Cell Transplantation Unit, HUCH Comprehensive Cancer Center, Helsinki, Finland; 11 Département d’Hématologie Clinique, CHU Lapeyronie, Montpellier, France; 12 Service des Maladies du Sang, CHRU, Angers, France; 13 EBMT Paris Study Office/CEREST-TC, Paris, France; 14 Hematology Division, Chaim Sheba Medical Center, Tel-Hashomer, Israel; 15 Adult Bone Marrow Transplantation Service, Department of Medicine, Memorial Sloan Kettering Cancer Center, 1275 York Avenue, Box 298, New York, NY 10065 USA

**Keywords:** Acute myeloid leukemia, T cell depletion, CD34-selected graft, Antithymocyte globulin, Allogeneic hematopoietic cell transplantation

## Abstract

**Background:**

Graft-versus-host disease (GVHD) is one of the leading causes of non-relapse mortality and morbidity after allogeneic hematopoietic stem cell transplantation (allo-HCT).

**Methods:**

We evaluated the outcomes of two well-established strategies used for GVHD prevention: in vivo T cell depletion using antithymocyte globulin (ATG) and ex vivo T cell depletion using a CD34-selected (CD34+) graft. A total of 525 adult patients (363 ATG, 162 CD34+) with intermediate or high-risk cytogenetics acute myeloid leukemia (AML) in first complete remission (CR1) were included. Patients underwent myeloablative allo-HCT using matched related or unrelated donors.

**Results:**

Two-year overall survival estimate was 69.9% (95% CI, 58.5–69.4) in the ATG group and 67.6% (95% CI, 60.3–74.9) in the CD34+ group (*p* = 0.31). The cumulative incidence of grade II–IV acute GVHD and chronic GVHD was higher in the ATG cohort [HR 2.0 (95% CI 1.1–3.7), *p* = 0.02; HR 15.1 (95% CI 5.3–42.2), *p* < 0.0001]. Parameters associated with a lower GVHD-free relapse-free survival (GRFS) were ATG [HR 1.6 (95% CI 1.1–2.2), *p* = 0.006], adverse cytogenetic [HR 1.7 (95% CI 1.3–2.2), *p* = 0.0004], and the use of an unrelated donor [HR 1.4 (95% CI 1.0–1.9), *p* = 0.02]. There were no statistical differences between ATG and CD34+ in terms of relapse [HR 1.52 (95% CI 0.96–2.42), *p* = 0.07], non-relapse mortality [HR 0.96 (95% CI 0.54–1.74), *p* = 0.90], overall survival [HR 1.43 (95% CI 0.97–2.11), *p* = 0.07], and leukemia-free survival [HR 1.25 (95% CI 0.88–1.78), *p* = 0.21]. Significantly, more deaths related to infection occurred in the CD34+ group (16/52 vs. 19/112, *p* = 0.04).

**Conclusions:**

These data suggest that both ex vivo CD34-selected and in vivo ATG T cell depletion are associated with a rather high OS and should be compared in a prospective randomized trial.

**Electronic supplementary material:**

The online version of this article (10.1186/s13045-018-0668-3) contains supplementary material, which is available to authorized users.

## Background

Allogeneic hematopoietic stem cell transplantation (allo-HCT) is the only potentially curative post-remission consolidation treatment for high-risk acute myeloid leukemia (AML) patients [1, 2]. However, preparative regimen-related toxicities and graft-versus-host disease (GVHD) have limited its widespread use. In particular, chronic GVHD (cGVHD) remains the leading cause of late non-relapse mortality (NRM) and morbidity after allo-HCT. Furthermore, the increasing use of G-CSF mobilized peripheral blood stem cells (PBSC) [3], a well-identified risk factor for chronic GVHD [4, 5], is associated with an increased incidence of cGVHD [3, 6]. Therefore, identification of the most effective prevention of GVHD is required to improve patients’ outcome after allo-HCT, particularly in the setting of PBSC transplantation.

In vivo graft manipulation with antithymocyte globulin (ATG) [7–13] or alemtuzumab [14] and ex vivo graft manipulation with CD34 selection and T cell depletion [15–19] are strategies that are associated with lower rates of chronic GVHD. Since 2000, five phase III randomized trials have investigated the efficacy of rabbit ATG for GVHD prophylaxis in patients who received myeloablative (MAC) allo-HCT from unrelated or HLA-identical matched donors [7–13]. In all these studies, the use of ATG was associated with a protective effect against cGVHD and in all but one study [12], overall survival (OS) and progression-free survival were not significantly affected [13]. Therefore, in vivo T cell depletion using ATG is now considered a standard for GVHD prevention after PBSC transplantation using related HLA-identical or unrelated donors in many centers. On the other hand, several studies have shown that the use of ex vivo T cell-depleted (TCD) grafts combined with ATG significantly reduces the risk of GVHD without the need for post-transplant immunosuppression [16, 20, 21]. Although several different approaches for T cell depletion of the allograft have been used over the years, more recently, removal of T cells from the graft has routinely been performed through positive selection of CD34+ cells using immunomagnetic beads [22]. To date, no study has compared outcomes in AML patients after myeloablative allo-HCT with ex vivo TCD using CD34 selection or in vivo TCD using ATG. To compare the efficacy of both approaches, we retrospectively evaluated the outcomes of patients with intermediate or high-risk AML in first complete remission (CR1) who underwent myeloablative allo-HCT with either in vivo TCD with ATG within the European group for Blood and Marrow Transplantation (EBMT) centers or ex vivo TCD CD34 selected (CD34+) graft at the Memorial Sloan Kettering Cancer Center (MSKCC).

## Methods

### Study design and data collection

This retrospective multicenter analysis was performed and approved by the Acute Leukemia Working Party (ALWP) of the EBMT group registry and the institutional review board of the MSKCC. A list of the EBMT participating centers is available online (Additional file 1). The study included all adult patients (age > 18 years) with AML, with intermediate or high-risk cytogenetic, in first morphological CR, who received an in vivo or ex vivo T cell-depleted myeloablative allo-HCT from an HLA matched related (MRD) or unrelated (UD) donor using a peripheral blood stem cell graft between 2005 and 2015. Cytogenetics were classified according to the European Leukemia Net [23]. All allografts were obtained from HLA-A-, HLA-B-, HLA-C-, and HLA-DRB1-matched donors. All patients underwent myeloablative conditioning. Patients at MSKCC received ex vivo TCD graft (CD34+ group, *n* = 162) after conditioning with one of the following preparative regimens as previously reported: (1) i.v. busulfan (Bu) 0.8 mg/kg/dose for 10 or 12 doses over a 4-day period, melphalan 70 mg/m^2^/day for 2 days, and i.v. fludarabine (Flu) 25 mg/m^2^/day for 5 days (*n* = 107); (2) hyperfractionated total body irradiation (TBI) 13.75 Gy over 4 days followed by i.v. thiotepa 5 mg/kg/day for 2 days and i.v. cyclophosphamide (Cy) 60 mg/kg/day for 2 days (*n* = 45) or i.v. Flu 25 mg/m^2^/day for 5 days (*n* = 10). Peripheral blood grafts underwent CD34 cell selection using the ISOLEX 300i magnetic cell selection system (Baxter, Deerfield, IL), followed by sheep red blood cell-rosette depletion (Isolex-E, *n* = 53); or CD34+ selection using the CliniMACS CD34 Reagent System (Miltenyi Biotech, Gladbach, Germany) (*n* = 109). The two approaches provide a similar level of T cell depletion (log10 5.3 with Isolex-E vs. 5.1 with CliniMACS, Jakubowski et al., in preparation). All patients received equine ATG (30 mg/kg total dose, *n* = 12) or rabbit ATG (thymoglobulin 5 mg/kg total dose, *n* = 143) to prevent graft rejection, except for those patients receiving a transplant from an HLA-matched related donor and conditioned with hyperfractionated TBI, thiotepa, and Flu (*n* = 7). No GVHD prophylaxis was administered post-transplantation.

Within the EBMT centers, patients received unmodified grafts and in vivo T cell depletion using rabbit ATG (group ATG, *n* = 363) after one of the following preparative regimens: (1) Bu 9.6–12.8 mg/kg total dose and i.v. fludarabine (*n* = 173), (2) Bu 9.6–12.8 mg/kg total dose and i.v. Cy 100–120 mg/kg total dose (*n* = 129), or (3) high-dose TBI and i.v. Cy 100–120 mg/kg total dose (*n* = 61). Patients received either thymoglobulin (*n* = 233) or grafalon (formerly ATG-fresenius, *n* = 130) for prevention of graft rejection and of GVHD. These patients received post-HCT GVHD prophylaxis consisting of cyclosporine alone (*n* = 62) or in combination with methotrexate (*n* = 213) or mycophenolate mofetil (*n* = 60), tacrolimus in combination with methotrexate (*n* = 2) or sirolimus (*n* = 10) or other combinations (*n* = 16).

Supportive care and antimicrobial prophylaxis were administered according to standard guidelines and include infection prophylaxis for *Pneumocystis jirovecii* and herpes virus. All patients were assessed at least once per week for cytomegalovirus (CMV) and Epstein-Barr virus (EBV) reactivation in the peripheral blood by polymerase chain reaction, to initiate preemptive therapy [24].

### Statistical analysis

Endpoints included OS, leukemia-free survival (LFS), cumulative incidence of relapse, NRM, acute and chronic GVHD, and refined GVHD-free relapse-free survival (GRFS) [25]. All outcomes were measured from the time of allo-HCT. OS was based on death, regardless of the cause. LFS was defined as survival with no evidence of relapse. OS and LFS rates were calculated by the Kaplan-Meier estimator. Cumulative incidence functions were used to estimate the probabilities of acute and chronic GVHD, NRM, relapse, and GRFS to accommodate competing risks. NRM and relapse were the competing risks for each other. Patients alive without relapse were censored at the time of last contact. For acute and chronic GVHD, the competing risk was death without the event. For refined GRFS, the events were relapse, grade III–IV acute GVHD, or extensive cGVHD and the competing risk was death without the events [25]. Acute GVHD was defined according to the standard criteria [26]. Due to the retrospective nature of this analysis, NIH cGVHD classification [27] was not available for EBMT centers; therefore, Shulman et al. classification (limited versus extensive) [28] was used for all patients.

Patients’ characteristics were compared between the CD34+ and the ATG groups using the chi-square test or the Fisher exact test for categorical variables and the Mann-Whitney test for continuous data. Univariate analyses were performed using the log-rank test for OS and LFS and Gray’s test for cumulative incidences. Chronic GVHD was analyzed as a time-dependent variable. For multivariate regression, a Cox proportional hazards model was built. Impact of age was analyzed per decade. Results were expressed as hazard ratio (HR) with 95% confidence interval (CI). All tests were two-sided and the type-1 error rate was fixed at 0.05. Statistical analyses were performed with SPSS 19 (SPSS Inc. /IBM, Armonk, NY) and R 3.0.1 (R Development Core Team, Vienna, Austria) software packages.

## Results

### Patient and donor characteristics

Patients and transplant characteristics are summarized in Table 1. Patients, in the CD34+ group who were significantly older, were more likely to have a matched related donor and a Karnofsky performance status < 90% compared to the ATG group. There were no statistically significant differences between groups regarding donor and patient gender, CMV serological status, and cytogenetic risk factor. The median follow-up among surviving patients was 35.4 (range, 2–139) months and was significantly longer in the CD34+ group, 58 (range, 6–139) months, compared to that in the ATG group, 24.5 (range, 2–131) months (*p* < 0.001). As a result, all patients were censored at 2 years for the comparison between groups.

**Table 1:** Study population characteristics

Characteristic (%)	ATG (*N* = 363)	CD34 (*N* = 162)	*p*
Patient age, median (range)	46 (19–77)	58 (20–73)	< 0.001
Year of transplant	2012 (2005–2015)	2011 (2005–2015)	
Patient gender			
Male	200 (55%)	90 (56%)	0.92
Female	163 (45%)	72 (44%)	
Female donor to male patient	65 (18%)	37 (23%)	0.19
Karnofsky performance scale			
≥ 90%	258 (76%)	100 (63%)	0.003
< 90%	83 (24%)	59 (37%)	
unknown	22	3	
CMV serologic status			
Seronegative donor-recipient pair	114 (32%)	49 (33%)	0.4
Time from diagnosis to transplant, months (range)	4.9 (1.9–14.3)	4.3 (1.7–11.4)	0.001
Cytogenetic			
Intermediate	259 (71%)	112 (69%)	0.61
Poor	104 (29%)	50 (31%)	
Donor			
Matched related donor	118 (33%)	73 (45%)	0.006
Unrelated donor	245 (67%)	89 (55%)	
Conditioning regimen			
Busulfan + fludarabine	173 (48%)	0	< 0.0001
Busulfan + cycloclophosphamide	129 (36%)	0	
TBI + cyclophosphamide	61 (17%)	0	
Busulafan + fludarabine + melphalan	0	107 (66%)	
TBI + cycloclophosphamide + thiotepa	0	45 (28%)	
TBI + fludarabine + thiotepa	0	10 (6%)	
GVHD prophylaxis			
CsA	62 (17%)	–	–
CsA + MMF	210 (58%)	–	
CsA + MTX	65* (18%)	–	
Tacrolimus + sirolimus	10 (3%)	–	
PT Cy	5 (1%)	–	
Others	11 (3%)	–	
Antithymocyte globuline			
Thymoglobulin (median dose, mg/kg; range)	233 (5; 5–7.5)	143 (5; 2–5)	
Grafalon, (median dose, mg/kg; range)	130 (35; 8–60)	0	
Equine ATG, (median dose, mg/kg; range)	0	12 (30; 15–30)	–
Cells doses			
CD34+ cells, × 10^6^/kg (range)	6.13 (1.64–21.2)	8.18 (1.1–31.2)	< 0.0001
CD 3+ cells, × 10^6^/kg (range)	213 (1.13–643)	0.002 (0–0.063)	< 0.0001

*Abbreviations*: *TBI* total body irradiation, *GVHD* graft versus host disease, *CsA* ciclosporine A, *MMF* mycophenolate mofetil, *PT Cy* post-transplant cyclophosphamide, *NA* not available

*In two patients, CsA have been substituted by tacrolimus

### Engraftment

Engraftment was achieved in 362/363 patients (99.7%) in the ATG and 159/162 (98.1%) in the CD34+ group (*p* = 0.06). The median time to neutrophil recovery was significantly longer in the ATG group: 16 (range, 6–34) days compared with 10 (range, 8–20) days in the CD34+ group (*p* < 0.0001).

### Overall survival and leukemia-free survival

**Fig. 1:**
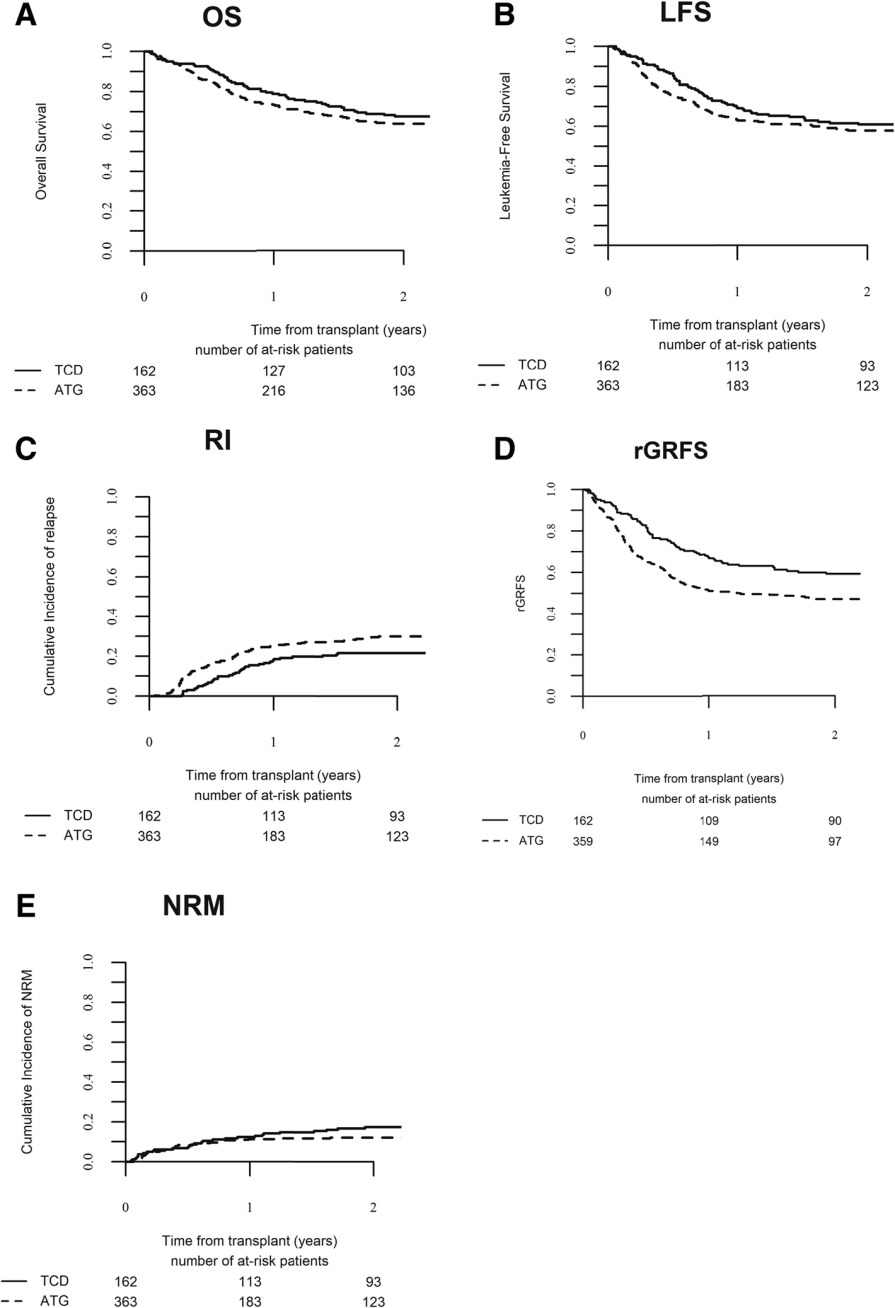
Outcome after allo-HCT. Overall survival (**a**), leukemia-free survival (**b**), cumulative incidence of relapse (**c**), cumulative incidence of GVHD-free and relapse-free survival (**d**), and cumulative incidence of non-relapse mortality (**e**). OS, overall survival; LFS, leukemia-free survival; RI, relapse incidence, rGRFS, refined GVHD-free relapse-free survival; NRM, non-relapse mortality

Univariate and multivariate analyses of transplantation-related events are summarized in Tables 2 and 3**,** respectively. The Kaplan-Meier estimate of OS at 2 years was 63.9% (95% CI, 58.5–69.4) in the ATG group and 67.6% (95% CI, 60.3–74.9) in the CD34+ group (*p* = 0.31, Fig. 1a). In multivariate analysis, there was no significant difference in OS between the TBI group and the Bu group (HR, 1.43; 95% CI, 0.97–2.11; *p* = 0.07). The only parameters with a significant impact on OS in multivariate analysis were patients’ age and cytogenetic status. OS was significantly lower in patients with poor as compared to intermediate-risk cytogenetics (HR, 1.16; 95% CI, 1.11–2.19; *p* = 0.009) and in older patients (HR, 1.15; 95% CI, 1.00–1.34; *p* = 0.049). The Kaplan-Meier estimate of LFS at 2 years was 57.9% (95% CI, 52.4–63.4) in the ATG group and 61.0% (95% CI, 53.4–68.5) in the CD34+ group (*p* = 0.29, Fig. 1b). In multivariate analysis, there was no significant difference in LFS between the ATG and the CD34+ groups (HR, 1.25; 95% CI, 0.88–1.78; *p* = 0.21). The only parameter with a significant impact on LFS in multivariate analysis was cytogenetic status. LFS was significantly lower in patients with poor compared to those with intermediate risk cytogenetics (HR, 1.70; 95% CI, 1.26–2.31).

**Table 2:** Transplant-related events univariate analysis

Characteristic (%)	ATG (*N* = 363)	CD34 (*N* = 162)	*p*
OS at 2 years, months (95% CI)	69.9 (58.5–69.4)	67.6 (60.3–74.8)	0.31
LFS at 2 years, months (95% CI)	57.9 (52.4–63.4)	61.0 (53.4–68.5)	0.29
Cumulative incidence of NRM at 2 years (95% CI)	12.1 (8.9–15.9)	17.4 (12.0–23.6)	0.16
Cumulative incidence of relapse at 2 years (95% CI)	30.0 (24.9–35,2)	21.6 (15.6–28.3)	0.03
Cumulative incidence of grade II–IV aGVHD at 100 days (95% CI)	21.2 (17.1–25.6)	11.3 (7.0–16.8)	0.006
Cumulative incidence of grade III–IV aGVHD at 100 days (95% CI)	6.2 (4–9.1)	1.3 (0.2–4.1)	0.01
Cumulative incidence of cGVHD at 1 year (95% CI)	27.6 (22.8–32.6)	2.5 (0.8–5.8)	< 0.0001
Cumulative incidence of extensive cGVHD at 1 year (95% CI)	11.3 (8.0–15.1)	2.5 (0.8–5.8)	0.001
Cumulative incidence of GRFS at 2 years (95% CI)	47.0 (41.4–52.5)	59.1 (51.5–66.7)	0.003
Cause of death			
GVHD	14 (12%)	5 (10%)	0.36
Infections	19 (17%)	16 (30%)	
Other	20 (18%)	7 (14%)	
Relapse/progression	59 (53%)	24 (46%)	

*Abbreviations*: *OS* overall survival, *CI* confidence interval. *LFS* leukemia-free survival, *NRM* non-relapse mortality, *aGVHD* acute graft-versus-host disease, *cGVHD* chronic graft versus host disease

**Table 3:** Transplant-related events multivariate analysis

Outcome	Hazard ratio (95% confidence interval)	*p* value
Overall survival		
ATG versus CD34	1.43 (0.97–2.11)	0.07
Age per 10 years	1.15 (1.00–1.34)	*0.047*
Poor versus intermediate cytogenetic	1.16 (1.11–2.19)	*0.009*
Unrelated versus related donor	1.23 (0.86–1.76)	0.27
Leukemia-free survival		
ATG versus CD34	1.25 (0.88–1.78)	0.21
Age per 10 years	1.03 (0.91–1.17)	0.60
Poor versus intermediate cytogenetic	1.70 (1.26–2.31)	*0.0006*
Unrelated versus related donor	1.22 (0.88–1.70)	0.23
Non-relapse mortality		
ATG versus CD34	0.96 (0.54–1.74)	0.90
Age per 10 years	1.32 (1.03–1.68)	*0.02*
Poor versus intermediate cytogenetic	1.08 (0.62–1.90)	0.77
Unrelated versus related donor	1.39 (0.8–2.42)	0.24
Relapse		
ATG versus CD34	1.52 (0.96–2.42)	0.07
Age per 10 years	0.93 (0.80–1.08)	0.31
Poor versus intermediate cytogenetic	2.10 (1.46–3.03)	*< 0.0001*
Unrelated versus related donor	1.10 (0.73–1.67)	0.63
Grade II-IV acute GVHD		
ATG versus CD34	2.05 (1.12–3.73)	*0.02*
Age per 10 years	0.99 (0.82–1.18)	0.87
Poor versus intermediate cytogenetic	0.90 (0.56–1.48)	0.66
Unrelated versus related donor	1.87 (1.12–3.09)	*0.02*
Chronic GVHD		
ATG versus CD34	15.07 (5.38–42.26)	*< 0.0001*
Age per 10 years	1.13 (0.94–1.36)	0.21
Poor versus intermediate cytogenetic	1.07 (0.66–1.74)	0.79
Unrelated versus related donor	1.58 (0.97–2.56)	0.07
GVHD-free relapse-free survival		
ATG versus CD34	1.60 (1.14–2.34)	*0.006*
Age per 10 years	1.01 (0.90–1.13)	0.88
Poor versus intermediate cytogenetic	1.65 (1.25–2.18)	*0.0004*
Unrelated versus related donor	1.42 (1.05–1.93)	*0.02*

*Abbreviations*: *ATG* antithymocyte globulin, *GVHD* graft-versus-host disease

### Relapse

At 2 years, the cumulative incidence of relapse was 30.0% (95% CI, 24.9–35.2) in the ATG group and 21.6% (95% CI, 15.6–28.3) in the CD34+ group (*p* = 0.03, Fig. 1c). In multivariate analysis, there was a trend toward a higher cumulative incidence of relapse in the ATG group (HR, 1.52; 95% CI, 0.96–2.42; *p* = 0.07). The only parameter with a significant impact on relapse incidence in multivariate analysis was cytogenetic risk status: relapse was significantly increased in patients with poor compared to intermediate risk cytogenetics (HR, 2.10; 95% CI, 1.46–3.03; *p* < 0.0001). Therefore, a subgroup analysis was performed to separately analyze patients with intermediate and high-risk cytogenetic. In patients with intermediate risk cytogenetic, the 2-year cumulative incidence of relapse was significantly higher in the ATG group compare to the CD34+ group [25.0% (95% CI, 19.5–30.6) versus 11.6% (95% CI, 6.5–18.4)]. In multivariate analysis, TCD approach was the only parameter with an impact on relapse with a significantly higher cumulative incidence of relapse in the ATG group (HR, 2.42; 95% CI, 1.22–4.78; *p* = 0.01). In contrast, in the subgroup of patients with high-risk cytogenetic, TCD approach has no impact on the cumulative incidence of relapse [42.7% (95% CI, 32.1–53.0) in the ATG group versus 44.0% (95% CI, 29.9–57.3)].

### Graft-versus-host disease

The day-100 cumulative incidence of grade II–IV and grade III–IV aGVHD were significantly higher in the ATG group, being 21.2% (95% CI, 17.1–25.6) and 6.2% (95% CI, 4–9.1), versus 11.3% (95% CI, 7.0–16.8) and 1.3% (95% CI, 0.2–4.1), respectively, in the CD34+ group (*p* = 0.006 and *p* = 0.01). In multivariate analysis, TCD approach was the only parameter with an impact on aGVHD. The cumulative incidence of grade II–IV aGVHD was significantly higher in the ATG group compared with the CD34+ group (HR, 2.05; 95% CI, 1.12–3.73; *p* = 0.02). At 1 year, the cumulative incidence of cGVHD and extensive cGVHD were significantly higher in the ATG group, being 27.6% and 11.3%, versus 2.5% and 2.5%, respectively, in the CD34+ group (*p* < 0.0001 and *p* = 0.001). In multivariate analysis, TCD approach was the only parameter with an impact on cGVHD. The cumulative incidence of cGVHD was significantly higher in the ATG group compared with the CD34+ group (HR, 15.07; 95% CI, 5.38–42.26; *p* < 0.0001).

### Graft-versus-host disease-free relapse-free survival

At 2 years, the cumulative incidence of GRFS was significantly lower in the ATG group, being 47.0% (95% CI, 41.4–52.5) versus 59.1% (95% CI, 53.5–68.5) in the CD34+ groups (*p* = 0.003, Fig. 1d). In multivariate analysis, GRFS was significantly higher in the CD34+ group compared to the ATG group (HR, 1.60; 95% CI, 1.14–2.23; *p* = 0.006). In addition, cytogenetic status and type of donor were also associated with lower GRFS in multivariate analysis.

### Non-relapse mortality

At 2 years, the cumulative incidence of NRM was 12.1% (95% CI, 8.9–15.9) in the ATG group and 17.4% (95% CI, 12.0–23.6) in the CD34+ group (*p* = 0.16, Fig. 1e). In multivariate analysis, there was no significant difference in NRM between the ATG and CD34+ groups (HR, 0.96; 95% CI, 0.54–1.74; *p* = 0.90). The only parameter with a significant impact on NRM incidence in multivariate analysis was patient age: NRM significantly increased in older patients (HR, 1.32; 95% CI, 1.03–1.68; *p* = 0.02). NRM was related mainly to infection (*n* = 35) and GVHD (*n* = 19), others causes being hemorrhage (*n* = 2), sinusoidal obstruction syndrome (SOS, *n* = 4), cardiac toxicity (*n* = 1), graft failure (*n* = 2), secondary malignancy (*n* = 2), others (*n* = 13), unknown (*n* = 3). Deaths related to infectious complication were significantly more frequent in the CD34+ group (16/28 versus 19/53 in ATG group, *p* = 0.045), while there was no difference between groups regarding death related to GVHD (5/28 in CD34+ versus 14/53 in ATG group, *p* = 0.41).

### Donor lymphocyte infusion

Similar proportions of patients received DLI post-transplant in both cohorts. In the ATG T cell depletion cohort, 38 (10.5%) patients received DLI for the following indications: mixed chimerism in 8 patients, relapse in 19 patients, and prophylaxis in 11 patients. In the CD34+ cohort, 19 (11.7%) patients received DLI. The indications were mixed chimerism in 9 patients, relapse in 7 patients, and infection in 3 patients (2 EBV and 1 HHV-6).

## Discussion

The optimal goal of allo-HCT is to mediate graft-versus-tumor effect to achieve cure, while sparing patients from severe comorbidity. Over the last decade, patients’ outcomes have improved significantly with an improved OS and decreased NRM [3]. However, despite this progress, there has been an increase in the incidence of cGVHD, the leading cause of late NRM and morbidity after allo-HCT [3, 6], associated with the use of unrelated donors and of PBSC grafts [4, 5]. Identification of the best strategy for cGVHD prevention is of the utmost importance, and while TCD may be a potential strategy, there remains a concern that it may be at the expense of an increased incidence of relapse. The primary objective of this retrospective study was to assess the relative efficacy of two TCD approaches, in vitro TCD using ATG, and ex vivo TCD with CD34 selection, and showed high OS and LFS with both approaches in patients with AML in CR1 transplanted with matched donors after a MAC regimen.

Furthermore, our study confirms that TCD is associated with a low incidence of severe acute and chronic GVHD, with a day 100 cumulative incidence of grade III–IV aGVHD of 6.2% and 1.3% and a 1-year cumulative incidence of extensive cGVHD of 11.3% and 2.5% in the ATG and the CD34+ groups, respectively. The results in the ATG groups are in accordance with previously published prospective randomized trials, with an incidence of grade III–IV aGVHD ranging from 2.4 to 11.7% and an incidence of extensive or moderate to severe cGVHD between 5 and 13% [8–10, 12]. Similarly, in the CD34+ group, the cumulative incidence of severe grade III–IV aGVHD and extensive cGVHD compares favorably to data available from clinical trials. In a prospective randomized trial of ex vivo TCD, Wagner et al. reported a cumulative incidence of grade III–IV aGVHD and overall cGVHD of 19% and 29% [29]; however, TCD in that study was limited to 1 to 2 logs depletion. More recently, with the TCD techniques utilized by the MSKCC group that allow 3 to 4 logs of T cell depletion, Devine et al. reported a cumulative incidence of grade III–IV aGVHD and extensive cGVHD of 4.5% and 6.8%, respectively, in a multicenter prospective phase 2 trial [15]. Overall progress in ex vivo TCD techniques have achieved a very low incidence of GVHD, leading to a significantly lower cumulative incidence of both acute and chronic GVHD in the CD34+ group, translating in a higher GRFS in those patients. It should be noted that the use of CD34 selection incorporates ATG, which is primarily used to promote engraftment by abrogating host T cells, but likely has an effect on residual donor T cells in the CD34-selected graft. This potentially contributes to the low risk of GVHD as well as delayed immune recovery [30]. Finally, use of a matched unrelated donor remains associated with an increase incidence of grade II–IV aGVHD, while there was a trend toward a higher incidence of cGVHD in multivariate analysis, highlighting that TCD, either ex vivo or in vivo, does not completely overcome HLA barrier.

The increased incidence of GVHD seen in the ATG group does not translate in a higher cumulative incidence in NRM in those patients. In fact, there was no difference in the incidence of death related to GVHD between groups (12% in the ATG versus 10% in the CD34 groups), while there was more death related to infections in the CD34 group: 30% versus 17% in the ATG group. Delayed immune recovery after CD34-selected TCD allo-HCT contributes to an increase incidence of infectious complications, which may result in a lack of improvement in OS despite the low incidence of GVHD, compared to unmodified grafts [18, 30–36]. However, the median age in the CD34+ group was over a decade older than the ATG group. Age is a known risk factor for GVHD, NRM and delayed immune reconstitution, and the results of the study need to be considered in the context of this significant age difference between the cohorts.

While use of TCD was thought to be associated with an increase incidence of relapse, relapse incidence remains limited in our study, and no difference between groups was observed in multivariate analysis. This observation is probably related to the intensity of the conditioning regimen administered. Indeed, while in four out of six studies that include exclusively RIC regimen, ATG was associated with an increased risk of relapse, in studies that include MAC or MAC and RIC regimens together, no increase in relapse risk was reported [37]. Similarly, the MSKCC group recently reported a lower incidence of relapse using CD34-selected TCD allo-HCT with MAC compared to RIC [38]. In our study, while there were some differences in conditioning regimen between the two groups, all patients received a MAC regimen. Therefore, the incidences of relapse observed in our study are in accordance with previously published studies, despite the inclusion of one third of patients with poor risk cytogenetic status. Unfortunately, due to its retrospective nature being registry-based study, molecular characteristics were not available for all patients and we were not able to refine our analysis based on these parameters. In addition, the inhomogeneity of ATG doses used in our study may have interfered with patients’ outcome. Indeed, while lower ATG exposure is thought to be associated with a higher risk of GVHD, high exposure may produce excessive T cell depletion, leading to delayed immune reconstitution with increased risk of relapse, but also higher NRM, mostly as a result of infections [13]. Therefore, Ayuk et al. reported that use of lower doses of grafalon (30 versus 60 mg/kg) was associated with a lower NRM but had no impact on chronic GVHD and relapse rate after MAC allo-HCT [39]. Furthermore, Locatelli et al. evaluated even lower doses of grafalon (15 versus 30 mg/kg) in a phase III trial and found that they were associated with an improved OS and progression-free survival without significantly increasing the incidence of GVHD [40]. Finally, ATG pharmacokinetics and pharmacodynamics may also have a significant impact on HCT outcomes. For example, while ATG dose is based on patients’ weight, Admiraal et al. recently developed a pharmacokinetic model where absolute lymphocyte count at time of ATG administration was the only relevant predictor for ATG pharmacokinetics in adults [41]. Development of such approaches may help to further improve ATG safety and tolerability.

Our study does have some limitations, due in part to its retrospective nature and potential differences in supportive care. One limitation of our study is that all patients from the CD34+ group were treated in a single center highly experienced in this approach, while the ATG group was constituted from a multicenter-based registry. However, inclusion criteria were defined in order to constitute a cohort of patient as homogeneous as possible, CR1 AML patients with a matched donor that received rabbit ATG and a Bu-Cy, Bu-Flu, or Cy-TBI MAC regimen, in order to overcome this limitation and to reduce bias due to disease status and donor type on disease outcome.

Another potential limitation is the broader applicability of CD34 selection given the fact that all patients in the CD34 group were from a single center highly experienced in this approach. However, this approach has previously shown similar results in a multicenter phase 2 trial [15]. Furthermore, a randomized multicenter phase 3 trial is currently comparing this approach to post-transplant cyclophosphamide or tacrolimus and methotrexate in patients with acute leukemia and MDS receiving a MAC transplant from an 8/8 HLA-matched related or unrelated donor (NCT02345850).

## Conclusions

Overall, our study shows that NRM, OS, and LFS are similar after ex vivo CD34-selected and in vivo ATG TCD MAC allo-HCT from related/unrelated donors in patients with AML in CR1 and intermediate/high-risk cytogenetic. Notably, the cumulative incidence of acute (total and severe) and chronic GVHD was higher after allo-HCT with ATG, leading to a lower GRFS in those patients. In contrast, stronger immunosuppression in the CD34+ group leads to a higher incidence of infectious related death. Given the high OS seen in patients with AML in CR1 with both approaches, they should be compared in a prospective randomized trial.

## Additional file

Additional file 1: List of EBMT contributing centers by decreasing number of patients enrolled in the study. (PDF 38 kb)

## Abbreviations

allo-HCT: Allogeneic hematopoietic stem cell transplantation
ALWP: Acute Leukemia Working Party
AML: Acute myeloid leukemia
ATG: Antithymocyte globulin
Bu: Busulfan
cGVHD: Chronic graft-versus-host disease
Cy: Cyclophosphamide
EBMT: European group for Blood and Marrow Transplantation
Flu: Fludarabine
GRFS: GVHD-free relapse-free survival
GVHD: Graft-versus-host disease
HLA: Human leukocyte antigen
HR: Hazard ratio
LFS: Leukemia-free survival
MAC: Myeloablative
MRD: Matched related donor
MSKCC: Memorial Sloan Kettering Cancer Center
NRM: Non-relapse mortality
OS: Overall survival
PBSC: Peripheral blood stem cells
TBI: Total body irradiation
TCD: T cell-depleted
UD: Unrelated donor
