# Supplementary material for: Ex vivo and in vivo T cell-depleted allogeneic stem cell transplantation in patients with acute myeloid leukemia in first complete remission resulted in similar overall survival: on behalf of the ALWP of the EBMT and the MSKCC
Source: J Hematol Oncol. 2018 Oct 20;11:127. doi: 10.1186/s13045-018-0668-3 (PMC6195954; doi:10.1186/s13045-018-0668-3)
Supplement: Supplementary file 1 — List of EBMT contributing centers by decreasing number of patients enrolled in the study. (PDF 38 kb) [file 13045_2018_668_MOESM1_ESM.pdf]

List of EBMT contributing centers by decreasing number of patients enrolled in the study (Nº preceding name is the EBMT center identification code):

230 Marseille [Paoli Calmettes]
202 Basel [202]
267 Pessac [H Haut-Leveque]
515 Helsinki [Univ Central H]
926 Montpellier [University]
650 Angers [CHRU]
209 Leuven [Univ H]
212 Stockholm [Univ H]
556 Budapest [National Med Ctr]
661 Rennes [H Sud/Pontchaillou]
251 Caen [Hopital, Hematol]
253 Nantes [Hotel Dieu]
277 Lille [H Claude Huriez]
283 Lund [Univ H]
658 Bergamo [Ospedale, ematol]
207 Paris [St Louis]
810 Freiburg [University]
160 Paris [H Necker]
289 Goeteborg [Sahlgrenska Univ H]
614 Hamburg [Univ H]
676 Vandoeuvre_Les_Nancy [H d`Enfants]
726 Liege [University]
163 Piacenza [Osp Civile]
225 Turku [University]
242 Santander [Valdecilla]
246 Rotterdam [Erasmus MC]
264 Poitiers [H La Miletrie]
295 Hannover [Medical Univ]
409 Petach-Tikva [Beilinson H]
731 Umeå [Univ H]
746 Tartu [Univ H]
775 Paris [St Antoine]
785 Homburg [Univ Saarland]
234 Brussels [St. Luc]
644 Vilnius [Santariskiy Kl]
659 Brest [C.H.R.U Brest]
705 Udine [Univ H]
161 Tel_Aviv [Sourasky]
257 Dublin [St James]
270 Grenoble [H A Michallon]
523 Nice [H de l`ARCHET I]

610 Bratislava [Univ H]
656 Prague [Ist Hematology]
672 Strasbourg [H Hautepierre]
718 Pilsen [Charles Univ H]
996 Antwerp_Edegem [UZA]
208 Zürich [208]
215 Brussels [Jules Bordet]
233 Besancon [H Jean Minjoz]
239 Utrecht [University]
240 Bologna [S Orsola-Malpighi]
248 Pescara [Osp Civile]
256 Kiel [UKSH]
258 Jerusalem [Univ Hadassah]
261 Geneva [261]
262 Paris [Pitie-Salpetriere]
266 Uppsala [Univ H]
273 Clermont-Ferrand [Jean Perrin]
302 Zagreb [Univ H Rebro]
311 Wiesbaden [Kl Diagnostik]
323 Murcia [V Arrixaca]
369 Beirut [American Univ]
389 Leipzig [Univ, Haemat/Oncol]
397 Riyadh [King Faisal]
539 London [St George`s]
565 Maastricht [Univ H]
574 Olomouc [Univ H]
589 Adana [Baskent Univ]
597 Brno [Univ H]
606 Cuneo [S Croce e Carle]
623 Verona [Policlinico]
624 Toulouse [H Purpan]
640 Ljubljana [Univ Med Ctr]
645 Marburg [Philipps Univ]
652 Tricase_(Lecce) [C Panico]
666 Villejuif [Gustave Roussy]
729 Hradec_Králové [Charles U H, Hem]
740 Linköping [Univ H]
754 Tel-Hashomer [Univ Adults]
759 Barcelona [H Univ Bellvitge]
792 Catania [Osp Ferrarotto]
807 Berlin [Charité Univ]
941 Rouen [Becquerel]
977 Limoges [CHRU]
